# Additive and Multiplicative Effects of Socially Stigmatized Identities Using Linear Regression to Model Effects on Self-Reported Overall Health as Reported in the All of Us Research Program: Quantitative Analysis

**DOI:** 10.2196/76037

**Published:** 2026-04-02

**Authors:** Katrine LeStage, Robert Vogel, Julia Moore Vogel

**Affiliations:** 1 Hoosic Valley Central School, Schaghticoke, NY, United States; 2 Department of Psychiatry, University of California, San Diego, San Diego, CA, United States; 3 Scripps Research Translational Institute, Scripps Research Institute, 10550 North Torrey Pines Road, SGM-PLAZA, La Jolla, CA, 92037, United States, 858 784 2275

**Keywords:** intersectionality, overall health, multiple marginalization, All of Us Research Program, quantitative analysis

## Abstract

**Background:**

Individuals with one or more socially stigmatized identities experience extensive health disparities, resulting in poorer health outcomes. However, most studies consider the effects of only individual stigmatized identities.

**Objective:**

We aimed to quantitatively estimate the additive and multiplicative effects of stigmatized identities on self-reported overall health.

**Methods:**

We used survey data from 387,411 participants in the All of Us Research Program, which has assembled a disease-agnostic cohort intended to reflect the US population, to statistically estimate the first- and second-order effects of 47 stigmatized identities on self-reported overall health. We used a linear model to estimate the effects of individual and pairwise stigmas on self-ratings of overall health.

**Results:**

We began by aiming to create cohorts for all 93 stigmatized identities previously found to affect health, of which 47 (51%) could be practicably examined. We first modeled individual stigmas alone to contrast the results with those that included both individual and pairwise stigmas. After using the false discovery rate to adjust for testing multiple hypotheses in the collective model, 29 individual and 116 pairs of stigmas had statistically significant effects on self-reported overall health. All significant individual effects were negative or neutral except for skin cancer. Those with the largest negative effect on self-rated overall health were difficulty walking or climbing stairs, unemployed or unable to work, difficulty with errands, and low educational attainment. Pairs of intersecting stigmas had a mix of negative and positive incremental effects, indicating that some stigmatized identities are negative modifiers, such as depression, and other combinations are less negative than the sum of their individual negative effects, such as having difficulty with multiple types of activities of daily living. The individual stigmas with the largest number of statistically significant stigma pairs were unemployed or unable to work (14/47, 30%); depression and low income (11/47 each, 24%); and difficulty walking or climbing stairs, cognitive difficulties, obesity, and skin cancer (8/47 each, 17%).

**Conclusions:**

Taken together, numerous pairs of stigmatized identities significantly affect self-reported overall health. While each stigmatization has both direct and indirect effects on health, the relative importance of direct and indirect effects will vary. Many of these are aligned with prior literature, and others warrant further exploration. While the large sample size of this study is a strength, we were unable to model higher-order intersectionality and encourage future research exploring this. The individual and pairwise identities with significant negative effects should be incorporated into research and clinical care by considering the multidimensionality of individuals and how that affects their overall health.

## Introduction

Individuals with socially stigmatized identities experience extensive health disparities [1,2]. This includes individuals with a race and/or ethnicity other than White; people with disabilities; lesbian, gay, bisexual, transgender, and queer populations; and people with low socioeconomic status, among many others. Individuals with multiple identities that are socially stigmatized report experiencing more discrimination and a lower quality of life [3,4], reflecting the connection between intersectionality and health disparities [5,6]. However, most studies of participants experiencing health disparities analyze each group in isolation. Ignoring the multifaceted nature of each individual can lead to incorrect conclusions [7]. Furthermore, the intersections of specific stigmatized identities, which we refer to subsequently as stigmatizations or stigmas, have not been quantified.

Reducing health disparities is a crucial worldwide effort, including through sustainable development goals such as the United Nations’ stated goal to “leave no one behind” [8,9], which aims to reduce disparities more broadly, including reducing poverty, discrimination, and barriers that prevent accessibility. Each of these factors is inextricably intertwined, as people experiencing discrimination [10-12], poverty [13,14], and reduced access to health care [15,16] have poorer health outcomes. Furthermore, each of these effects is multipronged. Race-based discrimination often leads to lower quality health care [17,18], delayed care [19], as well as chronic stress and inflammation [20]. Taken together, racism leads to poorer mental and physical health outcomes [10,20-22]. These stigmatizations can lead to shorter life expectancies. A study using neighborhood-level data from across the United States found that social determinants of health alone accounted for 60% of premature deaths in Chicago [23]. Once disparities are well understood, interventions can be designed to address the societal and structural barriers that are responsible for them [24].

To date, concomitant quantitative estimates of additive and multiplicative effects of stigmatized identities have been lacking. A lack of the requisite sample size has precluded such an analysis, given the need for large numbers (eg, over a thousand) of simultaneous statistical estimates under a linear model. To this end, we use the large-scale and diverse participant base of the All of Us Research Program, a large disease-agnostic study funded by the National Institutes of Health that aims to reflect the US population [25,26]. Participants were recruited from the United States, through physical sites and remote enrollment, and asked to complete surveys, share biospecimens, electronic health records, and wrist-worn wearable data. The data are curated and made available to qualified researchers through the Researcher Workbench [26]. We used this resource to analyze self-reported overall health data from all 387,411 participants with available data, enabling the analysis of 47 individual and 1124 pairs of stigmatized identities that were selected from a prior study defining stigmas that affect health [27]. We statistically estimated the additive and multiplicative effects of stigmatizations on self-reported overall health in an effort to identify any synergistic pairs that should be considered in the course of health research and care. The goal of this work is to quantitatively estimate the additive and multiplicative effects of stigmatized identities on self-reported overall health.

## Methods

### Data Source

We used data from the All of Us Research Program in which participants completed an online consent form and several surveys. The data for this manuscript were sourced from the Basics, Overall Health, and Personal and Family History surveys, all of which are publicly available on the program’s Researcher Workbench website [28].

### Ethical Considerations

The original data collection for this study was approved by the All of Us Research Program’s Institutional Review Board (IRB; reference number 2016-05), which governed the informed consent process. The study was reviewed by the Scripps IRB (reference number IRB-24‐8390) and determined to be exempt from human subjects research; therefore, consent was not required for this specific work. The data were accessed between April 5, 2023, and November 15, 2024; the data were deidentified [29]. Furthermore, we followed the All of Us Research Program’s Data and Statistics Dissemination Policy, which precludes disseminating information on groups of fewer than 20 participants [30]. However, poorly conducted research can cause harm beyond the direct study participants by drawing incorrect or even stigmatizing conclusions [31]. The All of Us Research Program requires all researchers accessing the Workbench to complete training on what qualifies as stigmatizing research and commit to not using program data to conduct stigmatizing research. Consequently, we have made every effort to use inclusive language that does not blame or stereotype the already stigmatized populations that are the topic of this study. Participants were offered US $25 compensation to offset the time and travel involved in participating in a blood draw, and some participants were offered additional compensation through pilots [32]; no compensation was offered for this specific study.

### Analysis Methods

We explored multiple potential methods to determine which method was best suited for these data and was feasible within computational constraints. The 3 methods we explored and did not move forward with were (1) ordered logistic regression, which did not converge; (2) LASSO (least absolute shrinkage and selection operator) multinomial regression, which resulted in a high level of sparsity that was not aligned by the underlying data; and (3) ordinal regression models with an elastic net penalty, which was too computationally intense when the regularization parameters were appropriately extended. We determined that using a linear model was most suitable for this initial exploration due to computational feasibility and interpretability. We estimated the effects of individual and pairwise stigmas on overall health self-ratings. Estimates of effect sizes under the model were computed by the *lm* linear regression tool in the R (R Foundation for Statistical Computing) statistical programming language. We eliminated collinear stigmatizations so that the matrix of covariates was of full rank; the two elements that were removed were subsets of other race or ethnicity categories. Cohort creation and data analysis were completed within the Researcher Workbench using responses to specific surveys. Figures were created in R Studio (version 2024.09.0+375; RStudio, Inc). Our code is available in Multimedia Appendix 1 and can be copied and rerun within the All of Us Researcher Workbench.

## Results

### Participants

This study included 387,411 participants from the All of Us Research Program, which has assembled a disease-agnostic cohort intended to reflect the US population and, for example, includes 45% individuals with a race and/or ethnicity other than White. Participants were recruited across more than 340 physical locations and online through health care provider organizations, including several federally qualified health centers, community partners, earned media, online recruitment campaigns, and educational tours throughout the country [25,26,33]. All participants completed an online consent process that included verifying their eligibility and viewing educational videos describing aspects of the program [34]. Considerable effort was made to enable access for any interested, nonincarcerated adult in the United States, particularly those who did not have access to health systems that traditionally lead health research. Focus was placed on recruiting individuals who have been historically underrepresented in biomedical research based on their race, ethnicity, educational attainment, income, location, and other factors [26]. All participants who had completed the relevant surveys and had their responses made available through the All of Us Researcher Workbench were included in this analysis.

### Stigmatized Identities

**Figure 1.**
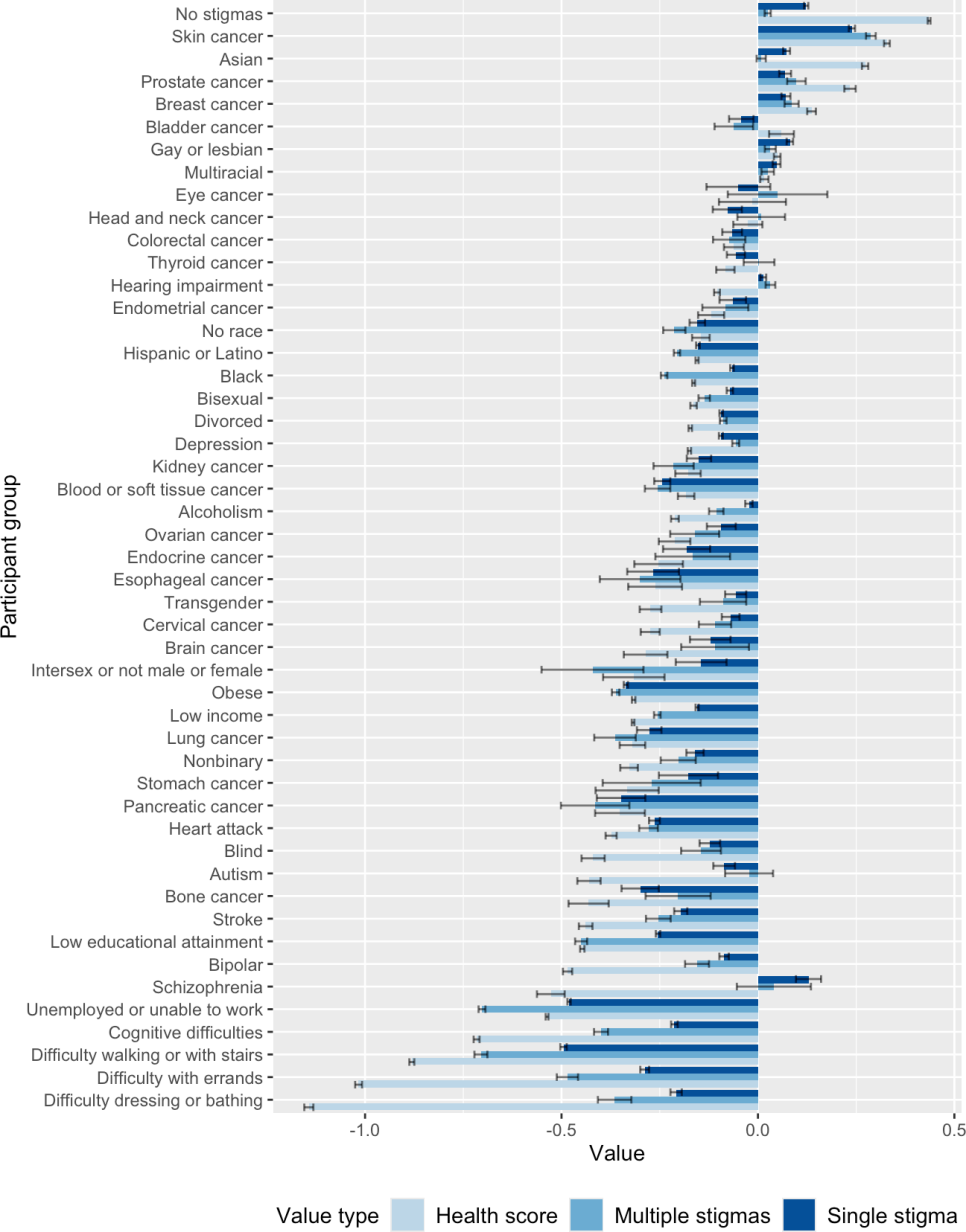
Values for the average overall health rating per stigma relative to the mean for the cohort as a whole (light blue), the estimated effect size of each stigma in the model with individual and pairwise effects (medium blue), and their estimated effect size in the model with only individual effects (dark blue). They are ordered from highest average overall health rating (top) to lowest (bottom). A value of “0” represents the average overall health value across the entire cohort, negative values represent a lower than average value, and positive values represent a higher than average value. A value of “1” represents 1 unit on the survey response scale (eg, a change from “good” to “fair” would be “–1”). The sample sizes for each individual stigmatized identity are included in Figure S3 in Multimedia Appendix 2.

We began by aiming to create cohorts for all 93 stigmatized identities that had previously been found to affect health [27]. We created cohorts for every stigmatization within the All of Us Researcher Workbench controlled tier (version 7) [35]. Of the 93 stigmatized identities identified, 47 (51%) were included in the analyses [27] because they met the minimum sample size of 150 participants. This included 20 (43%) types of cancer; 7 (15%) disability statuses; 5 (11%) lesbian, gay, bisexual, transgender, and queer categories; 4 (9%) individuals with a race and/or ethnicity other than White; 4 (9%) socioeconomic statuses; 4 (9%) miscellaneous medical diagnoses; and 3 (6%) mental health diagnoses (see Figure 1 for the full list). Between these categories, a variety of types of stigma have been reported; for example, some people avoid others with life-threatening illnesses such as cancer due to an unwillingness to consider their own risk of cancer [36]. Ableism can be rooted in similar concerns; lack of familiarity with certain types of disabilities; and harmful, ill-informed beliefs about the value of disabled people [37]. Each category has direct and indirect effects on health [38-42], which cannot be differentiated from each other in our analysis. For example, a direct effect might be a physiological impact of a diagnosis, and an indirect effect might include the social acceptability of that diagnosis, and whether the individual experiences increased discrimination, and subsequent downstream biological consequences [10]. All participants self-described their race or ethnicity, reflecting these categories as a social construct [43] and therefore should not be equated with genetic ancestry. Some of these identities have a direct effect on health (eg, types of cancer) and an indirect effect. We intentionally selected stigmatized identities that can be both transient (eg, low income) or permanent (eg, race and ethnicity), so that their individual effects could be modeled. Individuals who reported being White and not having any of the other stigmatized identities were included in the “no stigma” group.

### Data Selection

We compared the All of Us Research Program participants’ self-reported overall health data [44]. We started by examining responses to questions regarding overall health, physical health, mental health, and quality of life, and found an average correlation of 0.61 between responses for a given individual (Figure S1 in Multimedia Appendix 2). For simplicity and to preserve statistical power, we constrained further analysis to overall health scores. We examined the average self-reported overall health within individual and pairs of stigmatizations. Overall health self-ratings were responses to the question “In general, would you say your overall health is: Excellent, Very Good, Good, Fair, Poor” that were converted to integers.

### Single Stigma and Independent Results

Different types of difficulties with activities of daily living had the lowest average overall health self-ratings, followed by low educational attainment (Figure 1, light blue, and Figure S2 in Multimedia Appendix 2). Asian, multiracial, and gay and/or lesbian individuals as well as those with breast, bladder, or prostate cancer had higher self-ratings than the average for the cohort as a whole. Individuals without stigmatized identities had the highest self-reported overall health. Due to relatively large sample sizes (median 3756; Figure S3 in Multimedia Appendix 2), most individual stigmatizations had mutually exclusive SEs of the means.

### Single Stigmatizations and Individual and Collective Results

Next, we created a model to simultaneously estimate only the effect of individual stigmatizations (Figure 1, dark blue). Then, we created a model to simultaneously estimate the effect size of all single and pairwise stigmatizations (Figure 1, medium blue). There are notable differences between the two models; for example, difficulties with activities of daily living, low educational attainment, being intersex, being Black, and being Hispanic and/or Latino have stronger negative effects in the model that estimates the independent contribution of each stigmatization.

### Single and Pairwise Stigmatizations and Collective Results

**Figure 2.**
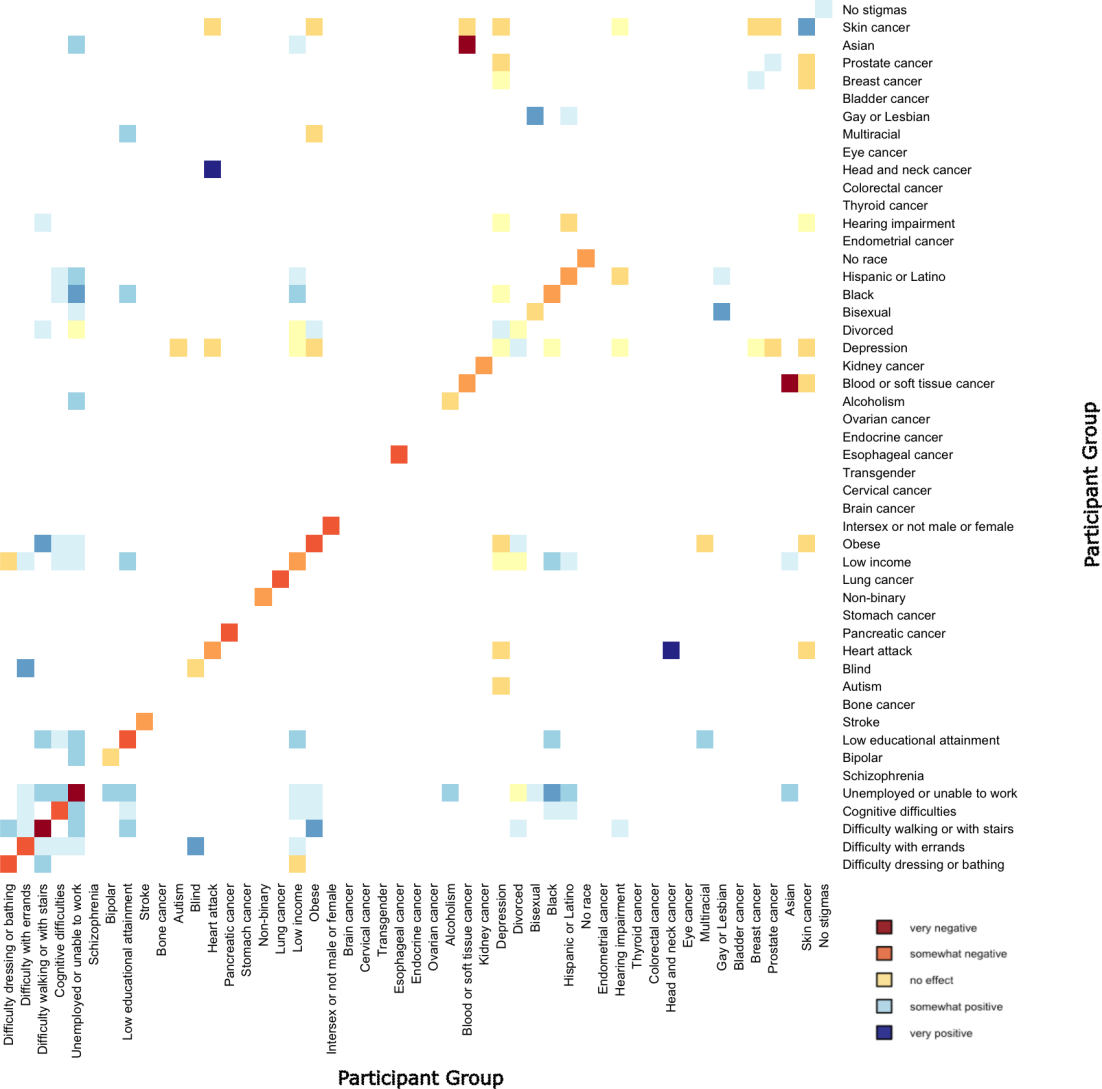
Results of the model of additive and multiplicative effects of socially stigmatized identities on self-reported overall health. This heat map shows the model estimates of the effect of individual (lower left to upper right diagonal) and pairwise (all other) stigmatized identities on self-reported overall health, sorted in the same order as in Figure 1. Each pair of labels meets at a small rectangle that is colored according to the effect of that pair of labels (or in the case that the two labels are the same, the single stigmatized identity). Red represents a negative effect on self-reported overall health, pale yellow represents a neutral effect, blue represents a positive effect, and white indicates that there were not enough data or the effect was not statistically significant. The number of participants in each group is mentioned in Multimedia Appendix 1.

When modeling both single and pairwise stigmatizations, the effect sizes of pairwise stigmatizations with individual effect sizes that were in the same direction and of relatively large magnitudes tended to have an opposite direction pairwise effect; this indicates the combined effect was less than the sum of the 2 individual effects. This is visualized with an enrichment of blue in the off-diagonal elements in the lower left of Figure 2 and an enrichment of red in the upper right, because the stigmatizations are ordered from the largest negative individual effect (left bottom) to largest positive individual effect (right top).

In the model that included multiplicative effects, out of 47 individual stigmatizations, 29 (62%) had statistically significant effect sizes, as did the group that declined to report their race or ethnicity (the largest effect sizes are listed in Table 1). When examining statistical significance, we used false discovery rate to adjust for testing multiple hypotheses [45] and considered *P* values less than .05 as statistically significant; note that exact *P* values are available in Multimedia Appendix 1. The 19 stigmatized identities that did not have statistically significant effects had a small effect size and/or a small sample size. Except for skin cancer, all significant individual effects were negative. Out of 1124 possible pairwise stigmas, 758 (67%) had sufficient sample sizes to estimate an effect size (with a requirement of at least 20 based on the All of Us Research Program policies [30]), and 116 (10%) had statistically significant effects. The individual stigmas with the largest number of statistically significant stigma pairs were unemployed or unable to work (14/47, 30%); depression and low income (11/47 each, 24%); and difficulty walking or climbing stairs, cognitive difficulties, obesity, and skin cancer (8/47 each, 17%).

**Table 1.** Individual stigmatized identities with the largest effects. This table lists the individual stigmatized identities with an effect size whose absolute value is greater than 0.15, ordered from the largest negative effect to the largest positive effect. This includes 6 cancer types; 5 disability statuses; 3 race or ethnicity categories; 2 lesbian, gay, bisexual, transgender, queer, and nonbinary identities; 2 socioeconomic statuses; 1 mental health diagnosis; and 3 miscellaneous health statuses.

Stigmatized identity	Estimate (SE)	Adjusted *P* value
Difficulty walking or with stairs	–0.705 (0.017)	<.001
Unemployed or unable to work	–0.702 (0.009)	<.001
Difficulty with errands	–0.485 (0.027)	<.001
Low educational attainment	–0.450 (0.015)	<.001
Intersex or not male or female	–0.421 (0.130)	.012
Pancreatic cancer	–0.414 (0.087)	<.001
Cognitive difficulties	–0.399 (0.018)	<.001
Difficulty dressing or bathing	–0.365 (0.043)	<.001
Lung cancer	–0.364 (0.053)	<.001
Obese	–0.362 (0.010)	<.001
Esophageal cancer	–0.300 (0.103)	.031
Heart attack	–0.279 (0.024)	<.001
Low income	–0.256 (0.008)	<.001
Blood or soft tissue cancer	–0.255 (0.032)	<.001
Stroke	–0.254 (0.031)	<.001
Black	–0.239 (0.008)	<.001
Kidney cancer	–0.215 (0.051)	<.001
No race reported	–0.213 (0.028)	<.001
Hispanic	–0.206 (0.008)	<.001
Nonbinary	–0.203 (0.045)	<.001
Bipolar	–0.155 (0.030)	<.001
Skin cancer	0.287 (0.012)	<.001

The pair with the largest negative estimated effect was Asian and blood or soft tissue cancer. Most other pairs with negative effects were cancers, combined with other types of cancer or heart attack, along with autism and depression, and prostate cancer and depression. The pairs with the largest positive estimated effect sizes were head and neck cancer and heart attack, as well as blindness and difficulty with errands. In pairs with a positive effect, most individual stigmas had negative effect sizes, indicating that the combined effect was less negative than the sum of the two individual effects.

## Discussion

### Findings in Context

These analyses demonstrated statistically significant effects of 29 individual and 116 pairs of stigmatized identities on an individual’s rating of their overall health. The analyses revealed a wide variety of unknown causes to be further studied and pairs of intersecting variables that should be included within the analyses of specific diseases to ensure that subgroup effects are accurately characterized. This information adds to the existing literature describing multiple marginalization contributing to health disparities, that is, that having multiple stigmatized identities is often worse for one’s health than having one stigmatized identity [2,46,47]. One major contributor to these outcomes is discrimination—both in general, which contributes to poorer health outcomes [3,4], and in health care specifically, where it can decrease the quality of care [11,48]. Furthermore, these data contribute to the literature that structural racism contributes to health inequities in the United States [49].

Each stigmatization has both direct and indirect effects on health. For example, a cancer diagnosis can directly affect overall health survey responses by affecting how highly an individual rates components such as their overall health and quality of life [50,51]; an indirect effect on overall health could result from reduced social support, which has been observed after cancer diagnosis [52,53]. The direct and indirect effects could not be disentangled in this study, and the relative importance of direct and indirect effects will vary; for example, a stigmatized identity that is based on a health condition will usually have a larger proportion of direct effects. The large negative estimated effect of low educational attainment is striking and indicates a need to redouble efforts to improve health literacy within this population [54]; for example, additional efforts could be made to improve access to safe, culturally competent health care (especially preventative care); and for health care providers to ensure their communications are accessible to their patients. In our analyses, depression alone does not substantially impact overall health in a negative or positive direction; however, it produces a synergistic negative effect with several other stigmatizations, including types of cancer; this should be considered in the clinical management of their co-occurrence. Furthermore, ableism in health care can result in discrimination during health care visits [55,56], underrepresentation of disabled individuals in health care professions [57,58], and inadequate access to health care [56,59,60].

Cultural components of self-rated overall health should be explored further to understand how they affect health care. For example, previous work has noted higher overall health self-ratings from Asian participants compared to White participants in the United States with similar characteristics [61], which correlates with the higher overall health scores observed here (Figure 1).

This study has several limitations. First, the data were collected from participants only in the United States. Second, only individual and pairwise stigmatization were considered. There are likely higher-order effects that could not be analyzed while maintaining adequate statistical power. We recommend that future studies endeavor to explore higher-order effects and consider simplifying some groups, such as collapsing cancer types into 1 variable with and without cancer. Third, we assumed a linear relationship between the stigmas and the reported overall health and used linear regression, which assumes a continuous variable, whereas we used integers within a limited range. Fourth, individuals who may be clinically evaluated to have equivalent overall health may self-report different ratings [62]. Fifth, there are cultural differences and other population-specific biases with self-reported health data [62]. Sixth, we were unable to eliminate confounders that we did not include in the model; however, we mitigated this risk by including as many relevant confounders as practicable and using the most inclusive cohort available. Seventh, we did not use an analytical technique that was specifically designed to measure intersectionality, such as multilevel analysis of individual heterogeneity and discriminatory accuracy or chi-squared automated interaction detector [63]. We encourage the exploration of these techniques in future research. Notably, although divorced was included as a stigmatized status, separated and single parent were not listed in the reference we drew stigmatized identities from, and including them may strengthen the analysis; a similar argument can be made for cisgender women. Finally, individuals may evaluate their health relative to their expectations, their lived experience, and those around them, all of which can impact health. However, in cases where an objective measure would report higher overall health than a self-reported measure, the difference remained significant, as perception of one’s health can affect health outcomes [64]. We recommend that future research includes cisgender women as a stigmatized identity, given the ample documentation of the health impacts of stigma this group faces [65-68].

This work also has limitations that apply to many efforts to quantify intersectionality [46] and self-reported data. First, it was limited to studying the stigmatizations for which there were sufficient data available, and half of the stigmatized statuses could not be included [27]. Second, the participant’s specific status was based on one point in time. The statuses may change over time, such as mental health diagnoses, disability statuses, and employment status. Finally, the encoding of all stigmatizations as binary variables, even those for which a larger range of options could be used (eg, educational attainment and income). These limitations can be addressed in future research, for example, by studying smaller subsets of intersectionality in greater depth and aspects of health that vary between stigmatizations (eg, physical vs mental health), and other causes of health disparities such as discrimination, access to health insurance, and access to care providers that share their race or ethnicity and have cultural competence.

### Conclusions

Taken together, numerous pairs of stigmatized identities significantly affect self-reported overall health and therefore should be considered in research and clinical care. We recommend that both researchers and health care providers consider observed and potentially unobserved intersectionality when evaluating factors that affect one’s overall health. Policymakers may consider strongly suggesting or even mandating training on the impacts of intersectionality and the collection of important variables in the course of research and health care. Furthermore, data on impactful socially stigmatized identities should be collected to help researchers and clinicians better understand their participants and patients.

## Supplementary material

10.2196/76037 Multimedia Appendix 1 Analytical source code, aggregate data, and *P* values for all statistical tests.

10.2196/76037 Multimedia Appendix 2 Other supplemental figures.

## Abbreviations

IRB: Institutional Review Board
LASSO: least absolute shrinkage and selection operator
