# Supplementary material for: Additive and Multiplicative Effects of Socially Stigmatized Identities Using Linear Regression to Model Effects on Self-Reported Overall Health as Reported in the All of Us Research Program: Quantitative Analysis
Source: JMIR Form Res. 2026 Apr 2;10:e76037. doi: 10.2196/76037 (PMC13046218; doi:10.2196/76037)
Supplement: Multimedia Appendix 2 [file formative-v10-e76037-s002.docx]

**Supplemental Figures**

**
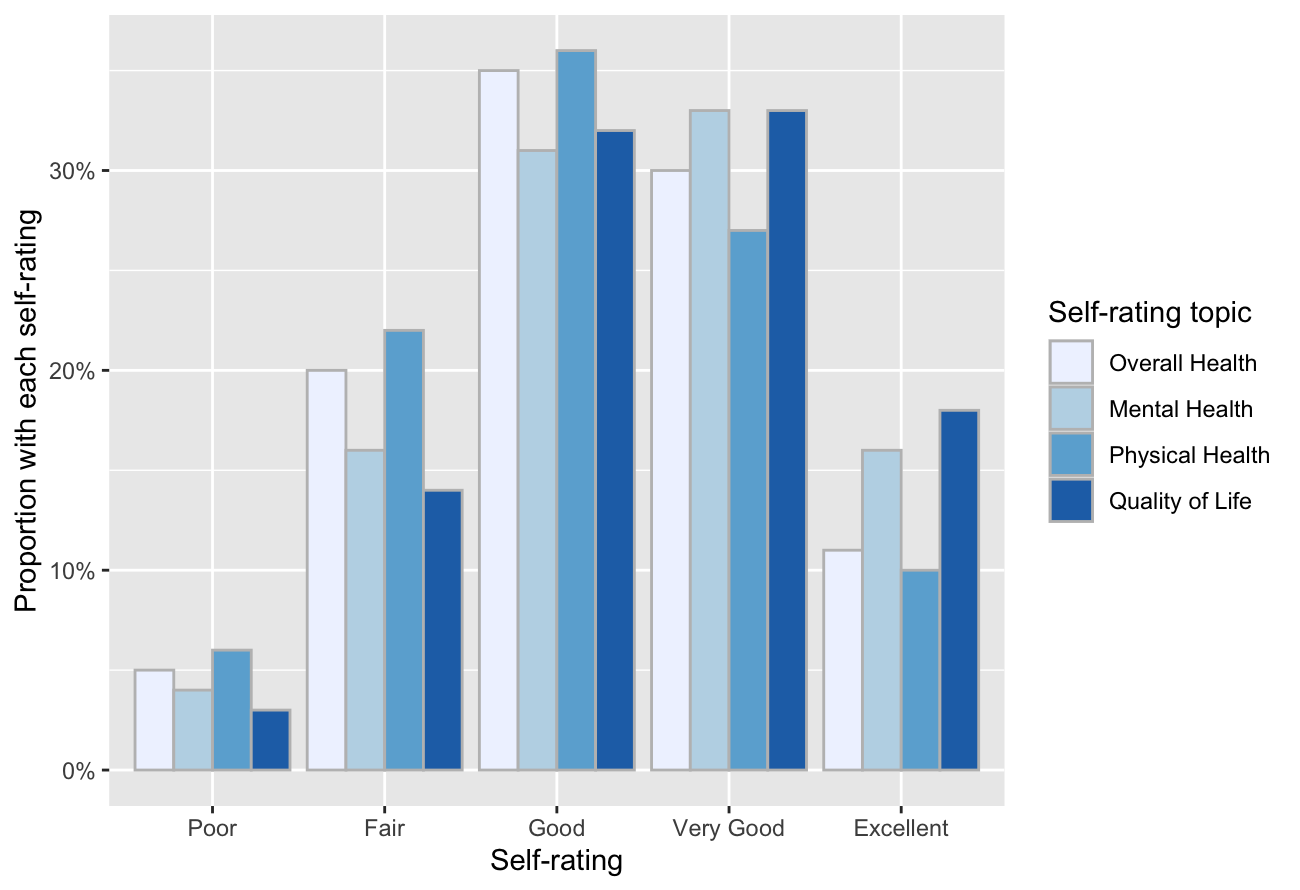
**

Figure S1. Self-ratings across dimensions of health. Because of the correlation between values (average 0.61), and the decrease in power that would have resulted from quadrupling the number of variables, we included only overall health in the model.

**
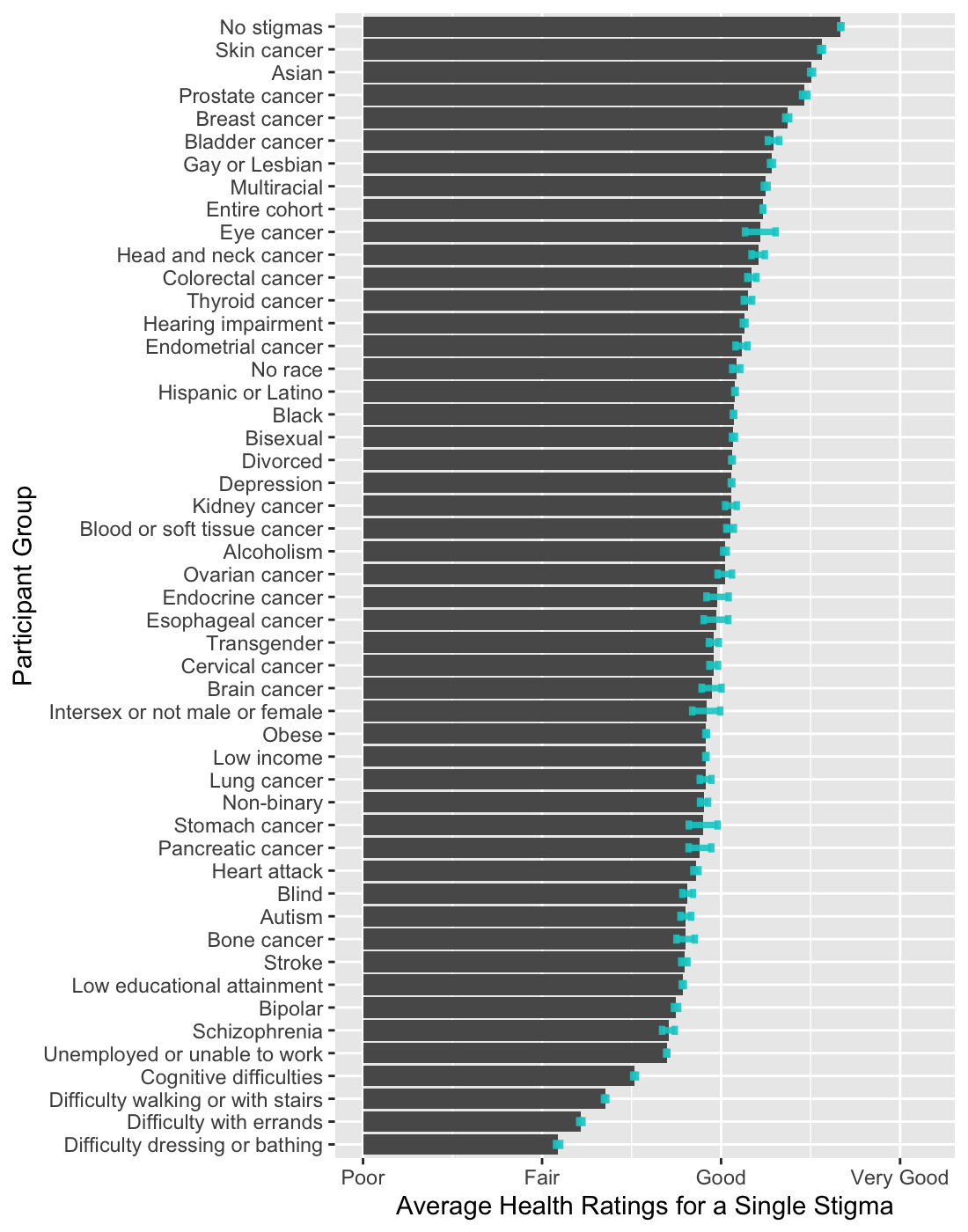
**


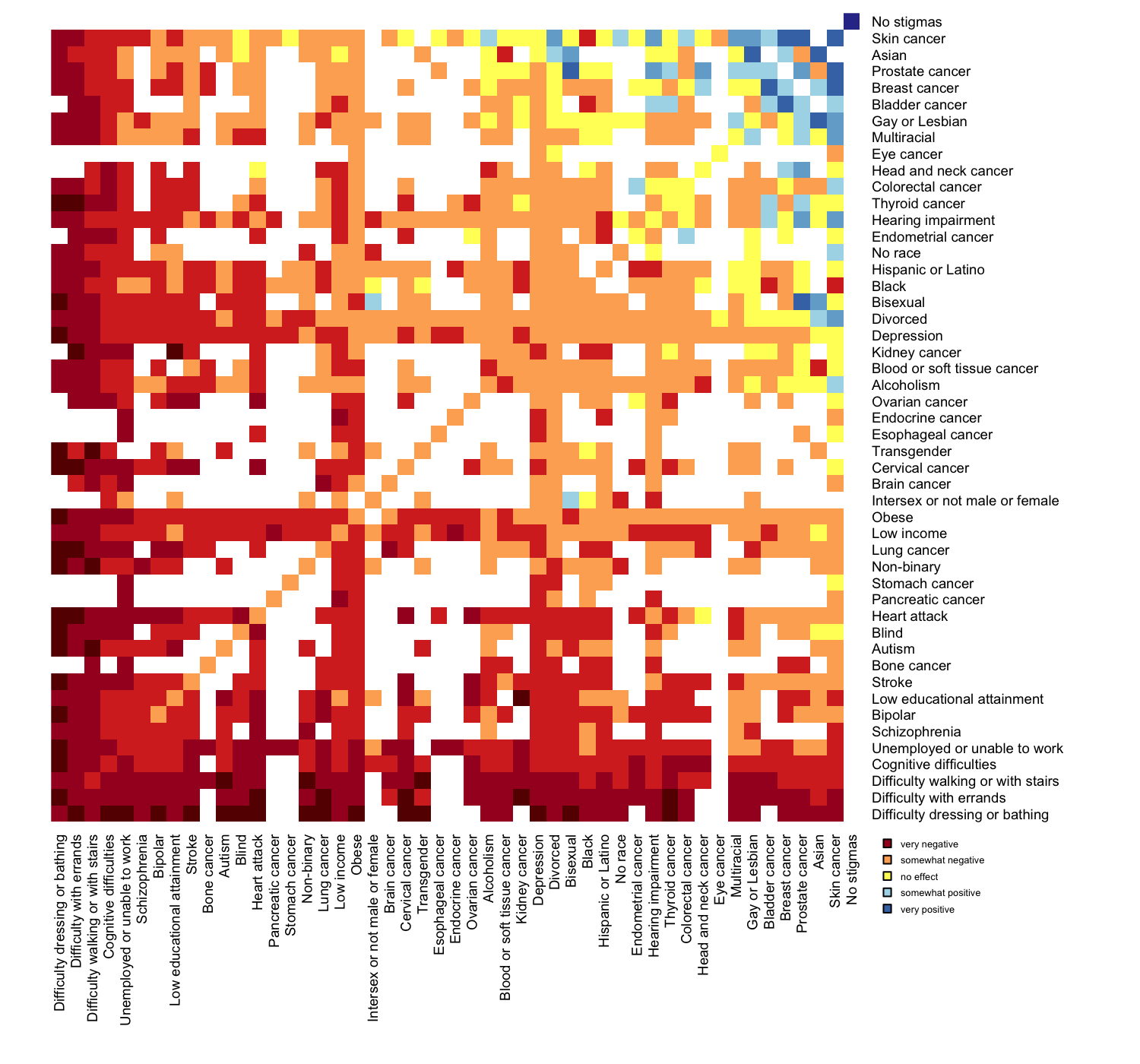


Figure S2. Average overall health ratings. Figure S2a: The average overall health rating and standard error of the mean of each individual participant group, sorted by estimated mean. Note, there is an option “Excellent” that is not listed because it is a larger value on the horizontal axis than any of the estimated means. Figure S2b: The average overall health rating for individual and pairwise stigmatizations, sorted by the estimated individual mean (visualized on the diagonal). Pairs with fewer than 20 participants are excluded, represented by white (as opposed to the pale yellow representing minimal effect relative to the average).


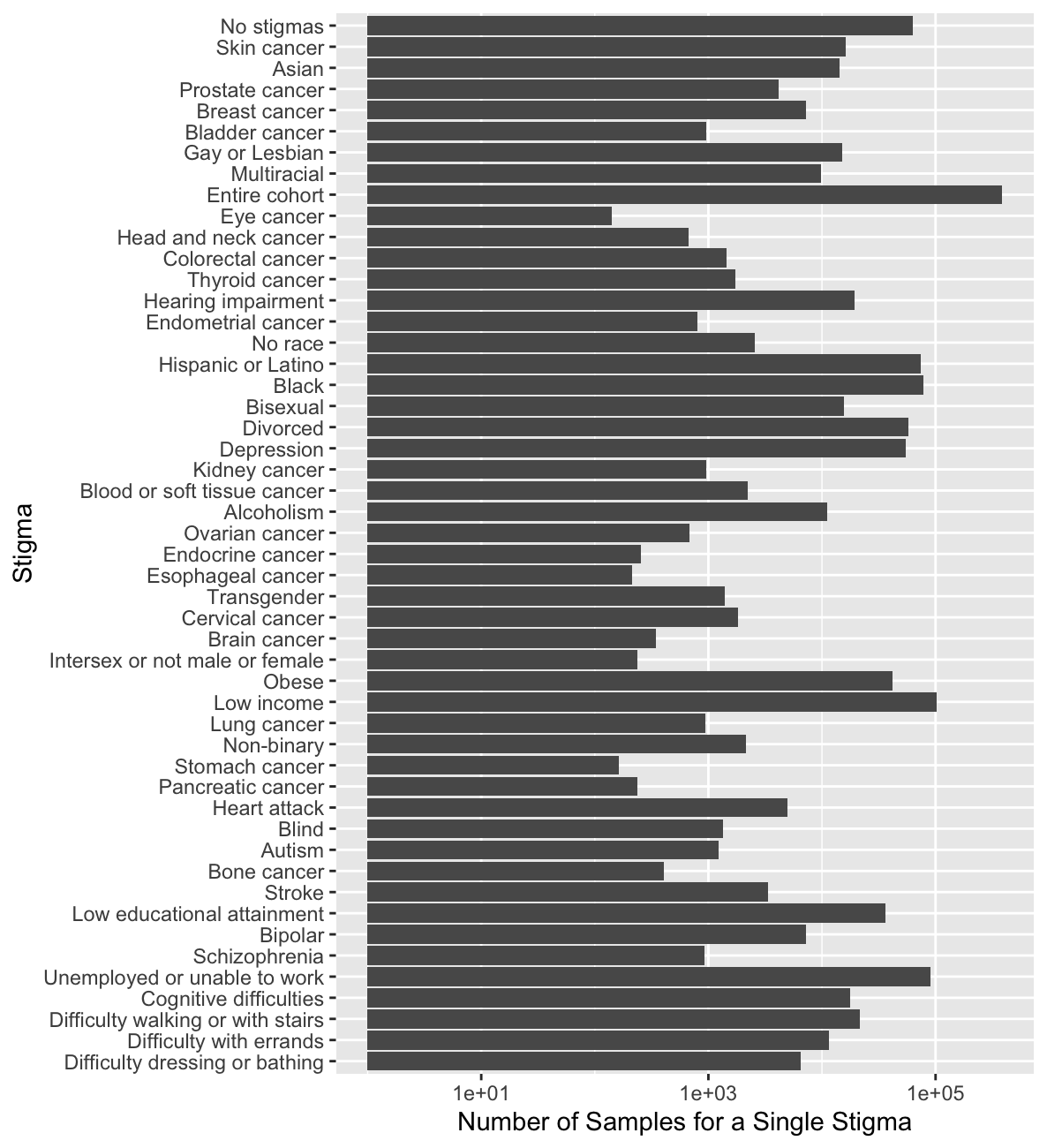


Figure S3. The number of participants with each individual stigma and the cohort as a whole; note the log scale. When comparing this to Figure 1, it is clear that stigmatizations with larger standard error of the mean generally have lower sample sizes.
